# Detection of human leptospirosis as a cause of acute fever by capture ELISA using a *Leptospira interrogans* serovar Copenhageni (M20) derived antigen

**DOI:** 10.1186/1471-2334-13-438

**Published:** 2013-09-20

**Authors:** Enrique Canal, Simon Pollett, Kristen Heitzinger, Michael Gregory, Matthew Kasper, Eric Halsey, Yocelinda Meza, Kalina Campos, Juan Perez, Rina Meza, Maruja Bernal, Alfredo Guillen, Tadeusz J Kochel, Benjamin Espinosa, Eric R Hall, Ryan C Maves

**Affiliations:** 1 U.S. Naval Medical Research Unit No. 6, Lima, Peru; 2 Sydney Institute of Emerging Infections and Biosecurity, University of Sydney, Sydney, Australia; 3 Institute of Tropical Medicine ‘Alexander von Humboldt’, Universidad Peruana Cayetano Heredia, Lima, Peru; 4 Universidad Nacional Federico Villarreal, Lima, Peru; 5 Naval Medical Research Center, Silver Spring, MD, USA; 6 Division of Infectious Diseases, Naval Medical Center, San Diego, CA, USA

**Keywords:** Leptospirosis, IgM, MAC ELISA, MAT, Peru

## Abstract

**Background:**

Leptospirosis is a potentially lethal zoonosis mainly affecting low-resource tropical countries, including Peru and its neighbouring countries. Timely diagnosis of leptospirosis is critical but may be challenging in the regions where it is most prevalent. The serodiagnostic gold standard microagglutination test (MAT) may be technically prohibitive. Our objective in this study was to assess the sensitivity, specificity, and predictive value of an IgM antibody capture enzyme-linked immunoassay (MAC-ELISA) derived from the M20 strain of *Leptospira interrogans* serovar Copenhageni (M20) by comparison to MAT, which was used as the gold standard method of diagnosis.

**Methods:**

Acute and convalescent sera from participants participating in a passive febrile surveillance study in multiple regions of Peru were tested by both IgM MAC-ELISA and MAT. The sensitivity, specificity, positive and negative predictive value (PPV, NPV) of the MAC-ELISA assay for acute, convalescent and paired sera by comparison to MAT were calculated.

**Results:**

The sensitivity, specificity, PPV and NPV of the MAC-ELISA assay for acute sera were 92.3%, 56.0%, 35.3% and 96.6% respectively. For convalescent sera, the sensitivity, specificity, PPV and NPV of the MAC-ELISA assay were 93.3%, 51.5%, 63.6% and 89.5% respectively. For paired sera, the sensitivity, specificity, PPV and NPV of the MAC-ELISA assay were 93.6%, 37.5%, 59.2%, 85.7% respectively.

**Conclusions:**

The M20 MAC-ELISA assay performed with a high sensitivity and low specificity in the acute phase of illness. Sensitivity was similar as compared with MAT in the convalescent phase and specificity remained low. Paired sera were the most sensitive but least specific by comparison to MAT serodiagnosis. NPV for acute, convalescent and paired sera was high. The limited specificity and high sensitivity of the MAC-ELISA IgM suggests that it would be most valuable to exclude leptospirosis in low-resource regions that lack immediate access to definitive reference laboratory techniques such as MAT.

## Background

Leptospirosis is a common and potentially lethal zoonotic disease with a world-wide distribution. It has a prevalence ranging from under 1 per 100,000 persons in developed temperate countries to over 100 per 100,000 persons in tropical developing regions [1]. As such, the burden of morbidity and mortality from leptospirosis occurs predominately in low-to-middle income countries in the tropics or subtropics [2].

*Leptospira interrogans*, the prinicipal pathogenic species of the genus, is an aerobic spirochete species with a wide range of animal reservoirs, including rodents and livestock [3,4]. Human inoculation via skin breaches or mucosal surfaces may occur upon exposure directly to animal urine or indirectly via contact with waste-contaminated water [5]. High risk groups for infection therefore may include veterinarians, abattoir workers, farmers and those living in riverside communities and areas of high rainfall [4,6]. In recent decades, outbreaks have been noted in adventure travellers, military personnel and triathlon competitors [1,7,8]. Leptospirosis may also cause significant morbidity in regions prone to flooding, even in developed countries with excellent healthcare infrastructure [9].

The clinical features of leptospirosis are often non-specific. Even with a compatible exposure history, the differential diagnosis may be broad [2,6]. Leptospirosis may progress rapidly from an apparently mild illness to life-threatening manifestations such as pulmonary haemorrhage, jaundice, acute kidney injury and meningoencephalitis. Such severe forms of leptospirosis have poor outcomes and carry a worse prognosis in those who do not receive prompt treatment [4].

For these reasons, a timely and accurate laboratory diagnosis is essential in the approach to suspected leptospirosis cases. This remains challenging, particularly in the very regions where the disease is most prevalent. Darkfield microscopy is inaccurate [2,3] and blood or urine cultures are slow and insensitive [3,4]. Newer molecular methods such as PCR and loop-mediated isothermal amplification are emerging as promising next-generation diagnostic tools but have not been widely validated. Moreover, their cost and technical expertise may inhibit their use in developing countries [5,10].

Serological methods remain the mainstay of diagnosis for leptospirosis [2,5]. Microagglutination testing (MAT) is still the most widely used serological assay and is regarded as the ‘gold standard’ of serodiagnosis, albeit with several limitations. The technical skill required for MAT limits its use in many non-reference laboratories, particularly in low-to-middle income countries [5]. Additionally, the need for a live panel of leptospires in MAT is a laboratory biohazard, and maintaining a geographically appropriate range of serovars may be challenging [11]. Other limitations include a limited sensitivity during the early phases of illness [3], inter-laboratory variation due to subjective interpretation of agglutination and difficulty in standardisation [3,6].

IgM antibody capture ELISA (MAC-ELISA) assays may offer a cheaper, simpler alternative method of serodiagnosis which can be standardised and may be more sensitive in the acute phase of illness. Other ELISA-IgM assays, including dipstick IgM, dot-ELISA IgM and commercial IgM ELISA have been validated in Asia, North America, Latin America and the Pacific [12-14]. However there is a paucity of validation studies carried out in Peru [15], a country which experiences a high burden of leptospirosis, with seropositivity rates ranging up to 28% in regions such as the Amazon basin [15].

The objective of this study was to assess the sensitivity, specificity and predictive value of an IgM antibody capture enzyme linked immunoassay (MAC-ELISA) derived from the (M20) strain of *Leptospira interrogans s*erovar Copenhageni M20 by comparison to MAT, which was used as the gold standard method of diagnosis.

## Methods

**Figure 1:**
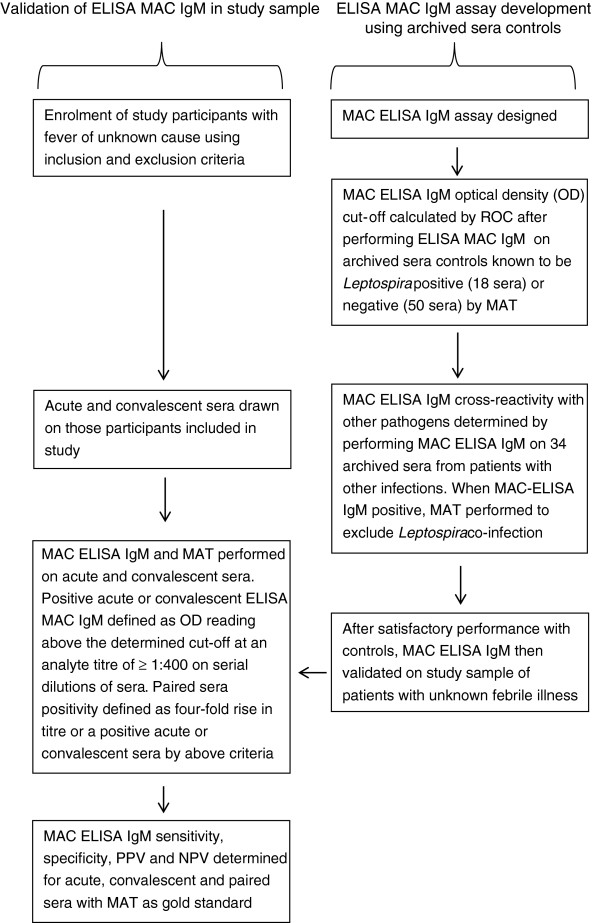
Overview of study methods.

An overview flowchart of the materials and methods is presented in Figure 1.

### Setting, study population and enrolment

In 1990, the United States Naval Medical Research Unit No. 6 (NAMRU-6) established a passive surveillance network for the study of acute febrile syndromes in the Peruvian Amazon, with expansion into Bolivia, Paraguay, and other regions of Peru in 2000. Specific details of this network have been previously described [16]. In this leptospirosis IgM MAC-ELISA validation study, patients aged five years or more with fever equal to or greater than 38.0°C for equal to or less than 7 days who presented for medical care at local hospitals or outpatient clinics in Peru (Iquitos, Yurimaguas, Madre de Dios, Junín, Tumbes) between January 2006 and February 2008 were invited to participate.

Patients were ineligible to participate if a clinically identifiable focus of infection was present. A clinically identifiable focus of infection included acute otitis media, sinusitis, purulent pharyngitis, cellulitis, acute urinary tract infection, dental caries, septic arthritis, pneumonia, pelvic inflammatory illness or peritonitis. Identification of malaria on blood films was not an exclusion criterion, as asymptomatic *Plasmodium* parasitaemia is well-described in endemic regions of Peru [17] and multiple etiologies of acute febrile illness have been detected in prior studies in the Amazon basin [18,19].

Study protocols (NMRCD.2000.0006 [Peru], NMRCD.2000.0008 [Bolivia], and NMRCD.2005.0008 [Paraguay]) were approved by the Naval Medical Research Center Institutional Review Board (Bethesda, Maryland, USA) in compliance with all U.S. Federal regulations governing the protection of human subjects. In addition, ethical review and approval was received from authorities in Peru (Dirección General de Epidemiología, Lima). Written consent was obtained from patients 18 years of age and older. For patients younger than 18 years, written consent was obtained from a parent or legal guardian. Additionally, written assent was required from patients between 8 and 17 years of age.

### Measures and procedures

Acute blood samples were obtained at the time of initial presentation for medical care by trained study personnel. Convalescent specimens were obtained between 10 and 21 days later. ‘Paired samples’ were defined as an acute and a convalescent sample of the same participant. Serum was separated from whole blood samples and divided into 4 aliquots, which were stored at -70°C. Samples were then transported in liquid nitrogen or on dry ice to Lima for testing by MAC-ELISA and MAT at the main NAMRU-6 laboratory in Lima and the Instituto de Medicina Tropical Alexander von Humboldt (Universidad Peruana Cayetano Heredia, Lima, Peru), respectively. Data on sociodemographic characteristics (namely age and sex) were collected by face-to-face interview with study personnel.

### Development of IgM MAC-ELISA assay

The IgM MAC-ELISA was developed using total antigen of *L*. *interrogans* serovar *Copenhageni* (M20). The antigen was developed in-house from culture specimens using a total sonicated extract at a concentration of 4300 ug/ml. This strain was chosen as previous ecological studies in Peru have implicated the *L*. *interrogans* serogroup Icterohaemorrhagiae (the same serogroup as *L*.*interrogans* serovar Copenhagi) as a common cause of human disease in this region [20]. A previous validation of a commercial *L*.*biflexa* antigen-based ELISA IgM assay in Peru showed a poor sensitivity and thus this species was avoided in the ELISA design [8].

Vinyl microplates were impregnated overnight with AffiniPure F(ab’)2 fragment goat anti-human IgM, Fc5μ in a 1:1000 dilution with PBS pH 7.4 (Jackson ImmunoResearch, West Grove, Pennsylvania, USA). Sera targets were diluted 1:100 with a sera diluting solution of phosphate-buffered saline (PBS), Tween 20 (Sigma-Aldrich, St Louis, Missouri, USA) and skim milk 5% (Difco), and incubated at 37°C for one hour before washing. M20 in-house antigen was used in a 1:800 dilution with sera diluting solution and incubated to 37°C for one hour before washing. Unconjugated hyperimmune rabbit anti-*Leptospira biflexa* IgG antibody (Accurate Chemical, Westbury, New York, USA) was used at a 1:2000 dilution with sera diluting solution and incubated at 37°C for one hour before washing. *L*. *interrogans* serovar Copenhageni (M20) rabbit IgG antibody was unavailable for use in this region at the time of the study. Goat anti-rabbit IgG (H + L)-HRP (Bio-rad, Hercules, New York, USA) was used as a conjugate at a 1:4000 dilution with sera diluting solution and incubated at 37C for one hour before washing. 2,2'-azino-di-3-ethylbenzthiazoline-6-sulphonate (KPL, Gaithersburg, Maryland, USA) was used as a chromogen and added to each well 15 minutes before reading optical density (OD) by the VMax microplate reader at 405 nm with a reference filter between 620 to 650 nm (Molecular Devices MDS Analytical Technologies, Sunnyvale, California, USA).

### Determination of MAC-ELISA optical density (OD) cut-off

OD cut-offs were determined using 50 banked *Leptospira*-negative control sera from healthy subjects from Lima, Peru and 18 banked positive control sera from Peruvian patients with known MAT-confirmed leptospirosis from the Instituto Nacional de Salud del Perú. These sera were not taken from the study sample, but were reference banked specimens. The cut-off point was calculated using the receiver operator characteristic curve (ROC) through the Data Statistical Package for the Social Sciences (Version 17.0, IBM, New York, New York, USA).

### Assessment for MAC-ELISA assay cross-reactivity with other pathogens

Archived banked sera specimens from 34 patients with evidence of other infections were also used as negative controls. These sera were not taken from the study sample, but were archived reference banked specimens. Sera from patients with a diagnosis of brucellosis (4 cases, diagnosis by in-house culture and indentification), bartonellosis (9 cases by in-house culture and *gltA gene* PCR assay [21], yellow fever (1 case by in-house anti-yellow fever IgM ELISA), dengue (1 case by in-house anti-dengue IgM ELISA), Oropouche virus (1 case by in-house anti-Oropouche IgM ELISA), Caraparo virus (1 case by in-house anti-Caraparo IgM ELISA), Venezualan Equine Virus (1 case by in-house anti-VEE IgM ELISA), *P*. *vivax* (1 case by Giemsa peripheral blood smear), *P*. *falciparum* (1 case by Giemsa peripheral blood smear), hepatitis A (3 cases by HAV-IgM ELISA by Beijing Wantai Biological Pharmacy Enterprise), hepatitis B (2 cases by Microparticle Enzyme Immunoassay, AxSYM Core, Abbott Laboratories, IL, USA), HIV (3 cases by Vironostika HIV-1 antigen by Biomerieux, Lyon, France; and INNO-LIA™ HIV I/II Score by Innogenetics, Gent, Belgium) and *Treponema pallidum* (6 cases by Rapid Plasma Reagin positive, confirmed by SYPHAGEN *Treponema pallidum* Hemaglutination Biokit/PRP NOSTICON II, Biomérieux, Lyon, France) were tested by the leptospirosis MAC-ELISA assay to assess for cross-reactivity between leptospirosis and these other pathogens.

### Sample testing by IgM MAC-ELISA

Acute, convalescent and paired sera samples were tested by IgM MAC-ELISA assay at serial dilutions in order to determine a quantitative measurement of IgM. Positivity of acute and convalescent sera was defined as an ELISA MAC IgM OD greater than the determined cut-off at titres of ≥ 1:400. Positivity of paired sera was defined by having a four-fold or greater increase in IgM titre, or by an IgM-reactive acute or convalescent sample at a titre of ≥ 1:400.

### Sample testing by MAT

MAT was performed at the Instituto de Medicina Tropical Alexander von Humboldt (Universidad Peruana Cayetano Heredia, Lima, Peru) as previously described with modifications based on local leptospirosis epidemiology [22]. A panel of 23 strains was employed: *L*. *interrogans* serovar Australis (Ballico), *L*. *interrogans* serovar Autumnalis (Akiyami), *L*. *borgpetersenii* serovar Ballum (Mus 127), *L*. *borgpetersenii* serovar Ballum (S602), *L*. *interrogans* serovar Bataviae (Van Tienen), *L*. *interrogans* serovar Bratislave (Jez Bratislava), *L*. *interrogans* serovar Canicola (Hond Utrecht IV), *L*. *weilii* serovar Celledoni (Celledoni), *L*. *interrogans* serovar Copenhageni (M20), *L*.*kischneri* serovar Cynopteri (3522 C), *L*. *interrogans* serovar Djasiman (Djasiman), *L*. *santarosai* serovar Georgia (LT 117), *L*. *interrogans* serovar Grippotyphosa (Moskva V), *L*. *borgpetersenii* serovar Hardjo(Hardjoprajitno), *L*. *interrogans* serovar Icterohaemorrhagiae (RGA), *L*. *borgpetersenii* serovar Javanica (Veldrat Batavia 46), *L*. *interrogans* serovar Mankarso (Mankarso), *L*. *noguchii* serovar Panama (CZ214K), *L*. *interrogans* serovar Pomona (Pomona), *L*. *interrogans* serovar Pyrogenes (Salinem), *L*. *borgpetersenii* serovar Tarassovi (Perepelitsin), *L*. *interrogans* serovar Wolfii (3705) and *L*. *licerasiae* serovar Varillal (Var 010). For acute and convalescent sera, 50% cell agglutination for one or more serovars at a dilution of ≥ 1:400 was considered positive for this region. For paired sera, a positive result was defined as a four-fold increase in titre between acute and convalescent samples, or an acute or convalescent sample with a titre of ≥ 1:400.

### Validation of IgM MAC-ELISA

Acute and convalescent sera were tested by both IgM MAC-ELISA and MAT, the latter of which served as the gold standard for leptospirosis positivity. All IgM MAC-ELISA and MAT data were entered into an electronic database and analysed using STATA 12 (Stata Corp, College Station, Texas, USA). The sensitivity, specificity, and positive and negative predictive values (PPV, NPV) of MAC-ELISA IgM for acute, convalescent and paired sera samples were calculated.

## Results

The OD cut-off value of the MAC-ELISA assay was established at 0.299 with 100% sensitivity and 98% specificity under the ROC curve. When tested against negative control sera with other infections for cross-reactivity, one HIV-positive serum (and none of the remaining 33 negative control sera) tested positive to leptospirosis by MAC-ELISA assay. This HIV-positive serum was subsequently determined to be leptospirosis-negative by MAT testing, thus excluding leptospirosis co-infection.

63 participants from the study sites were enrolled for validation of the MAC-ELISA IgM assay between January 2006 and February 2008. Sociodemographic characteristics of these participants are presented in Table 1. Of these participants, 20.6% (13/63) were positive by MAT on acute sera, 47.6% (30/63) were positive on convalescent sera and 49.2% (31/63) were positive on paired sera. 54.0% (34/63) were positive by MAC-ELISA IgM on acute sera, 69.8% (44/63) were positive on convalescent sera and 77.8% (49/63) were positive on paired sera. 44.4% (28/63) of patients were negative by MAT and MAC ELISA IgM on acute sera. 26.9% (17/63) of patients were negative by MAT and MAC ELISA IgM on convalescent sera. 19.0% (12/63) of patients were negative by MAT and MAC ELISA IgM on paired sera. The sensitivity, specificity, PPV and NPV of the MAC-ELISA assay for acute, convalescent and paired sera by comparison to MAT are presented in Table 2.

**Table 1:** **Sex**, **location and age of study participants**

	***n***	**%**
Total Participants	63	100
Sex		
Male	42	66.7
Female	21	33.3
Location (district, town^a^)		
Junin	3	4.8
Madre de Dios	1	1.6
Tumbes (Tumbes)	4	6.3
Loreto (Yurimaguas)	9	14.3
Loreto (Iquitos)	46	73
Age (years)		
Median	22	
Range	9 - 58	

^a^Town unknown in some participants.

**Table 2:** **Accuracy of *L***. ***interrogans *serovar Copenhageni MAC ELISA IgM in diagnosis of leptospirosis compared to microscopic agglutination testing in acute**, **convalescent and paired sera**

	**Sensitivity%**	**Specificity%**	**PPV%**	**NPV%**
	**(CI 95%)**	**(CI 95%)**	**(CI 95%)**	**(CI 95%)**
ELISA IgM,	92.3	56.0	35.3	96.6
Acute	(85.7 - 98.9)	(43.7 - 68.3)	(23.5 - 47.1)	(92.05 - 100)
ELISA IgM,	93.3	51.5	63.6	89.5
Convalescent	(87.2 - 99.5)	(39.2 - 63.9)	(51.8 - 75.5)	(81.9 - 97.1)
ELISA IgM,	93.6	37.5	59.2	85.7
Paired	(87.5 - 99.6)	(25.5 - 49.5)	(47.1 - 71.3)	(77.1 - 94.4)

*MAC-ELISA*, IgM antibody capture enzyme-linked immunoassay.

*NPV*, Negative Predictive Value, *PPV*, Positive Predictive Value.

## Discussion

The study sample was predominantly young (median age = 22), comparable to other studies in developed and developing countries [15,23]. The majority of cases were located in the Loreto district, an Amazonian region which has high rainfall and suitable ecology for leptospirosis. The town of Iquitos in particular is known to have a notably high seroprevalence of leptospirosis in slum areas [15]. As such, these study findings have reasonable generalizability to other regions of the world which suffer a high burden of *Leptospira* disease.

Our MAC-ELISA assay performed with a high sensitivity and low specificity in the acute phase of illness. Sensitivity was similar as compared with MAT in the convalescent phase and specificity remained low. Paired sera were the most sensitive but least specific by comparison to MAT serodiagnosis. Negative predictive value for acute, convalescent and paired sera was over 85%.

The sensitivity of this M20 MAC-ELISA IgM assay in the acute phase, while high, may have been limited by fundamental differences in MAT and ELISA techniques: MAT detects both anti-*Leptospira* IgM and IgG [5], whereas the MAC-ELISA detects solely IgM, and some patients with prior exposure to *Leptospira* may develop an early anamnestic IgG response. The limited specificity of this MAC-ELISA assay may also be attributable to persistent IgM circulation in those with recent or even remote leptospirosis exposure. High titres of IgM may be present in previously infected individuals even years after the infection [6,11]. This could potentially explain some of the MAT-positive/MAC ELISA-negative results in acute phase sera [11].

Cross-reaction of our ELISA IgM assay with a leptospirosis-negative/HIV-positive serum was identified in this study. HIV is one of the known pathogens that may cross-react with leptospirosis on ELISA-IgM testing, in addition to the other diseases included in our negative control panel. Such cross-reactivity to non-leptospirosis disease is a known drawback of ELISA-based assays [3]. The World Health Organisation (WHO) recommends confirmatory testing by MAT for this reason, although MAT may cross-react with certain other pathogens also [3,24]. Pathogen cross-reactivity could also have contributed to the low specificity shown with our MAC ELISA-IgM assay, particularly as the list of non-*Leptospira* infections in the exclusion criteria was not exhaustive.

Previous validation studies of other leptospirosis ELISA-IgM assays have shown variable sensitivities and specificities. In Peru, an investigation of an outbreak of leptospirosis in military recruits that used a commercial ELISA IgM assay (PanBio Leptospira IgM ELISA, PanBio, Queensland, Australia) demonstrated a paired sera sensitivity and specificity of 26% and 60%, respectively, when compared to MAT [8]. In patients with MAT or culture confirmed leptospirosis in Barbados, the PanBio ELISA-IgM assay demonstrated a sensitivity and specificity of 87.5 and 96.4% in the acute phase of illness. The same study evaluated a second commercial ELISA-IgM assay (InDx IVD ELISA, Integrated Diagnostics, Baltimore, MD, USA) with a sensitivity and specificity of 89.6 and 92.7% in the acute phase of illness [12]. An evaluation of IgM dot-ELISA dipstick, dipstick IgM assay and indirect haemagglutination assay by comparison to MAT, culture or tissue immunohistochemistry was performed on sera from febrile patients in North America, Hawaii, Puerto Rico, Palau and Thailand. In the acute phase, all three methods performed poorly with sensitivities of 50.0, 52.7 and 38.5% respectively. Overall paired sera specificities were 98.8, 89.6 and 95.8% respectively [13].

Comparison of the performance of other ELISA-IgM assays with this ELISA M20 MAC IgM assay is limited by inter-laboratory variation in ‘gold standard’ serodiagnosis (MAT) due to subjectivity in interpretation of agglutination [12]. Moreover, there is considerable debate in the literature as to what constitutes a positive MAT titre, even in leptospirosis-endemic countries. MAT titre cut-offs from 1:100 to 1:1600 have been used in the literature [2,25].

There were other limitations in this study, beyond the assumption that MAT is a ‘gold standard’ method of diagnosis. Additional limitations to this study include delayed seroconversion after leptospirosis, which has been well described. As many as 10% of patients will fail to seroconvert within 30 days of the onset of symptoms [3], and such cases may have been missed by either method of serodiagnosis in this study. Finally, *L*. *interrogans* serovar Copenhageni (M20) rabbit IgG antibody was unavailable for use in this region at the time of the study, and this would have been the ideal antibody to use in the ELISA design.

## Conclusion

The limited specificity and high sensitivity of the MAC-ELISA IgM suggest that it would be most valuable as a ‘rule-out’ test, with confirmation of a positive test needed by the more specific MAT at a reference laboratory, as per the most recent WHO technical guidelines on the diagnosis of leptospirosis [24]. Thus, in developing countries such as Peru, where access to reference serodiagnostic assays like MAT may be difficult in many regions and limited resources could preclude the purchase of commercial ELISA assays, this in-house ELISA IgM assay may be useful to ‘rule-out’ a diagnosis of leptospirosis in the early stages of febrile illness where timely initiation of anti-leptospiral antibiotics may be critical [13].

This study highlights the limitations of using whole-cell based enzyme immunoassays for leptospirosis diagnosis. Rather than using total sonicated antigen, extraction of particular candidate structural antigens, such as glycolipoprotein antigens, may improve specificity while preserving sensitivity [25]. Recent advances in genomic, proteomic, microarray and in-*silico* technologies may also aid in the design of future generation serodiagnostic assays for this common and potentially fatal infection [5,26-28].

## Competing interests

The authors declare that they have no competing interests.

## Authors’ contributions

EC, RCM, MG, MK, EH, BE, AG, SP and TJK contributed to study concept, protocol, design and logistics. EC, MG, RM, MB, KC, YM executed laboratory analyses. EC, RCM, SP, MG, MK, JP and KH managed and analysed data. EC, SP, RCM, MK and KH drafted and developed manuscript. All authors reviewed, read and approved the final manuscript.

## Disclaimer

The views expressed in this article are those of the authors and do not necessarily reflect the official policy or position of the Department of the Navy, Department of Defense, nor the U.S. Government. Several of the authors are U.S. Government employees or military service members. This work was prepared as part of their official duties. Title 17 U.S.C. §105 provides that ‘Copyright protection under this title is not available for any work of the United States Government.’ Title 17 U.S.C. §101 defines a U.S. Government work as a work prepared by a military service member or employee of the U.S. Government as part of that person’s official duties.

## Pre-publication history

The pre-publication history for this paper can be accessed here:

http://www.biomedcentral.com/1471-2334/13/438/prepub
